# Artificial Intelligence in Nursing Education: A Bibliometric Analysis

**DOI:** 10.1155/nrp/1538554

**Published:** 2026-02-11

**Authors:** Xiaoyue Yu, Weizhen Wu, Jun Ni, Zhifeng Lin

**Affiliations:** ^1^ Department of Obstetrics and Gynecology, Department of Nursing, Guangdong Provincial Key Laboratory of Major Obstetric Diseases, Guangdong Provincial Clinical Research Center for Obstetrics and Gynecology, Guangdong-HongKong-Macao Greater Bay Area Higher Education Joint Laboratory of Maternal-Fetal Medicine, The Third Affiliated Hospital, Guangzhou Medical University, Guangzhou, Guangdong, China, gzhmc.edu.cn; ^2^ Department of Outpatient, Kuangquan Street Community Health Service Center, Yuexiu District, Guangzhou, Guangdong, China; ^3^ Department of Nursing, Traditional Chinese Medicine Hospital of Liwan District, Guangzhou, Guangdong, China

**Keywords:** AI, artificial intelligence, database, hotspot, nursing students

## Abstract

**Background:**

The swift advancement of artificial intelligence (AI) has significantly impacted multiple industries. The prospects of AI within nursing education are especially promising, as it presents avenues for enhancing the preparation of forthcoming nursing practitioners in a constantly changing healthcare landscape.

**Methods:**

We employed VOSviewer, CiteSpace, and the Bibliometrix R package to effectively visualize the bibliometric findings.

**Results:**

A total of 430 publications were identified. The predominant sources of these publications were the United States of America and China, while the National University of Singapore emerged as the foremost contributor. The journals that provided the most substantial input in this area *included Nurse Education Today* and *Nurse Education in Practice*. Furthermore, keywords showed significant citation bursts and trending topics, such as technology, educational programs, and simulation.

**Conclusions:**

The results of this investigation offer novel perspectives for scholars who are keen on examining the integration of AI in the training of nursing education.

## 1. Introduction

The swift advancement of artificial intelligence (AI) has significantly impacted numerous industries [1–4], including finance, education, and transportation. This progress has resulted in remarkable advancements in efficiency and innovation. AI includes a variety of technologies [5–7], such as robotics, natural language processing, and machine learning, which have been applied across numerous fields. Furthermore, the healthcare industry has experienced a significant increase in the use of AI technologies, which are revolutionizing patient care and clinical practices [8, 9]. The potential of AI in nursing education is particularly encouraging, as it offers opportunities to improve the training of future nursing professionals in a rapidly evolving healthcare landscape [10, 11].

Nursing education plays an important role in developing a skilled nursing workforce that can meet the challenges of today’s healthcare environment [12, 13]. Healthcare systems worldwide face rising patient demands, a shortage of qualified nursing professionals, and the necessity for better patient outcomes. In this context, incorporating AI into nursing programs becomes increasingly important [14, 15]. Nurses play a vital role in the delivery of patient care, serving as primary caregivers between patients and the healthcare system [16]. However, although they hold critical position, they encounter various difficulties, such as heavy workloads, emotional strain, and the continuous requirement for professional growth [17–19]. By utilizing AI technologies, nursing education can improve the training and preparation of students. This equips students to effectively manage these challenges and thrive in their professional roles [13].

AI applications in nursing education are both diverse and innovative, featuring tools such as virtual simulations, personalized learning experiences, and intelligent tutoring systems [10, 20, 21]. Virtual simulations offer nursing students the chance to participate in authentic clinical scenarios, which considerably improve their decision‐making abilities and critical thinking. Furthermore, these simulations mitigate the risks linked to actual patient encounters [22]. A study on virtual patients in nursing education demonstrated that virtual patients have potential as an innovative assessment tool, allowing experienced nurses to refine their hypotheses and make informed clinical decisions [23]. Similarly, virtual patient simulations have been employed in psychiatric care to support collaborative learning, helping students overcome anxiety and fear associated with clinical practices [24]. Intelligent tutoring systems offer customized feedback and support, enabling students to identify areas needing improvement and to reinforce their understanding of the material [25]. A pilot study of an adaptive learning platform in a graduate nursing pathophysiology course demonstrated that 86% of participants strongly agreed they learned better with the platform [26]. Moreover, personalized learning approaches allow nursing students to advance at their own pace, accommodating their unique learning styles and needs [27]. These AI‐driven tools not only enrich the educational experience but also enhance students’ specific clinical skills, ultimately contributing to improved patient care.

To understand the future directions and current landscape of AI in nursing education, bibliometric analysis proves to be a valuable research method [28]. This quantitative approach allows for the examination of patterns and trends within academic literature. It provides insights into how knowledge in this field has developed and spread. By identifying key publications, influential authors, and emerging themes, bibliometric analysis sheds light on the research status of AI in nursing education and identifies areas that need further exploration. As the integration of AI continues to evolve, conducting a bibliometric analysis becomes crucial for recognizing existing gaps and opportunities. Therefore, this analysis can guide researchers and educators in shaping the future of nursing education in light of technological advancements in AI.

## 2. Method

In this investigation, we accessed the Web of Science Core Collection (WoSCC) database to retrieve literature. The search covered the period from January 1, 2012, to December 31, 2024, with data retrieval completed on April 18, 2025. The inclusion criteria were specifically tailored to capture only articles and reviews published in English that directly related to the role of AI in nursing education. Specifically, non‐English publications were excluded. Records that were not classified as articles or reviews, as well as those that did not focus on the field, were also excluded. The detailed search criteria and workflow are illustrated in Table S1 and Figure 1(a).

FIGURE 1(a) Flowchart outlining the methodology of the study. (b) Yearly research output in the field of nursing education. (c) Geographic distribution of research contributions categorized by region.

**Figure:**
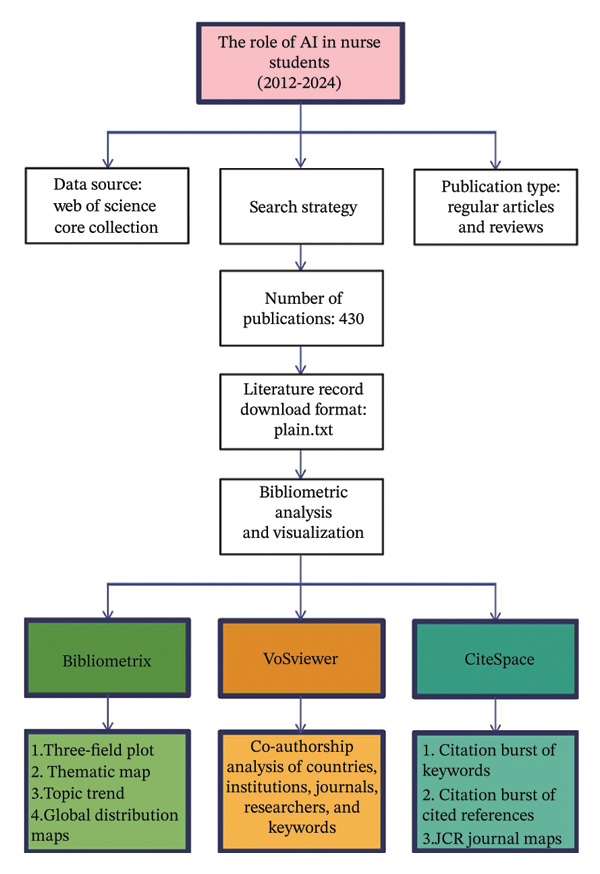
(a)

**Figure:**
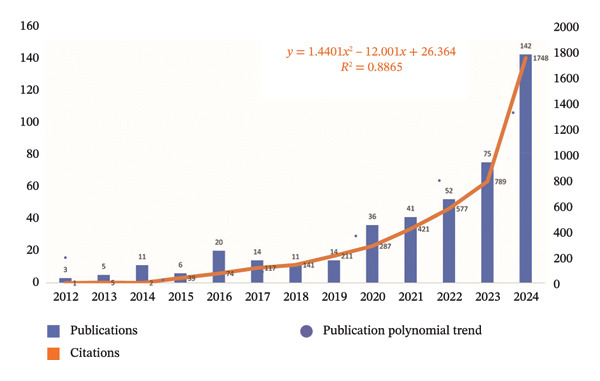
(b)

**Figure:**
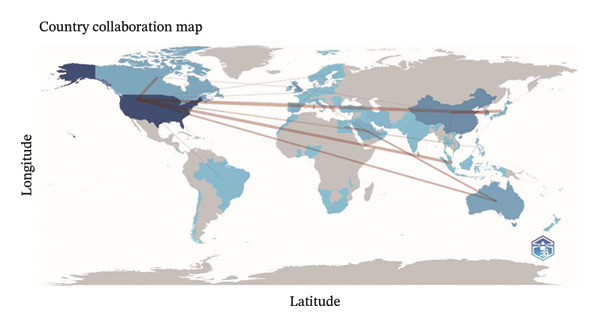
(c)

To improve the visualization of our bibliometric results, we utilized several tools, including VOSviewer (Version 1.6.20), CiteSpace (Version 6.4 R1), and the Bibliometrix R package [29–32]. VOSviewer was used to build co‐authorship, co‐occurrence, and co‐citation networks. CiteSpace identified key references and keywords with citation bursts (*γ* = 1 and time slice = 1) and created dual‐map overlays to show citation connections between journals. Furthermore, Biblioshiny, an online tool integrated within the Bibliometrix R package, was used to create global distribution maps, three‐field plots, and topic trends.

### 2.1. The Ethics Declaration

This study is based on publicly available datasets and does not need approval from an ethics committee.

## 3. Result

### 3.1. Literature Overview

A thorough analysis was conducted on 430 publications, which included contributions from 68 different countries, 775 institutions, and 1754 researchers. These publications were published in 194 distinct journals (Table S2). Figure 1(b) illustrates the patterns of annual publications from 2012 to 2024. Importantly, there was a significant rise in the number of publications since 2020, which suggested a rising academic interest in this particular field.

### 3.2. Analysis of Countries and Institutes

In the analysis of international collaboration networks, Figures 1(c) and 2(a) revealed the leading countries in research contributions and showed a map of country collaborations. The United States, China, and England emerged as the top contributors, with 138, 53, and 34 publications, respectively. Figure 2(b) and Table S3 showed the National University of Singapore as the most prolific, with 12 publications and 432 citations. Furthermore, Figure 2(c) indicates the distribution of core topics linked to each institution and country, highlighting the relationships and keyword distribution across the domain. Nearly all institutions and countries focused on the seven topics represented by the keywords. Notably, the United States and China made substantial contributions across all these topics.

FIGURE 2(a) Network of collaborations among countries. (b) Overall network map of institutions. (c) Three‐field plot. (d) Collaboration map of journals. (e) Density visualization of journals. (f) Collaboration map of co‐cited journals. (g) Density visualization of co‐cited journals. (h) A dual‐map overlay demonstrating the interconnections among various journals.

**Figure:**
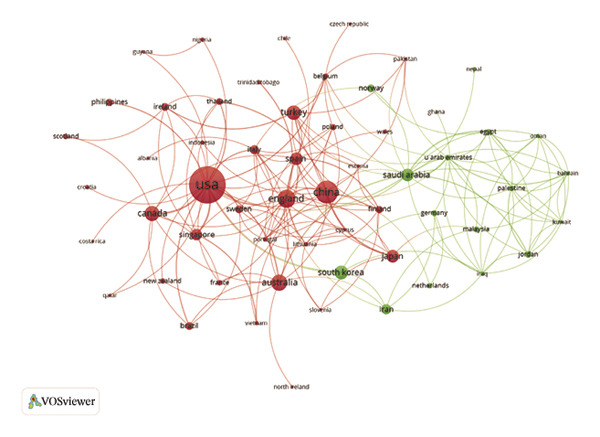
(a)

**Figure:**
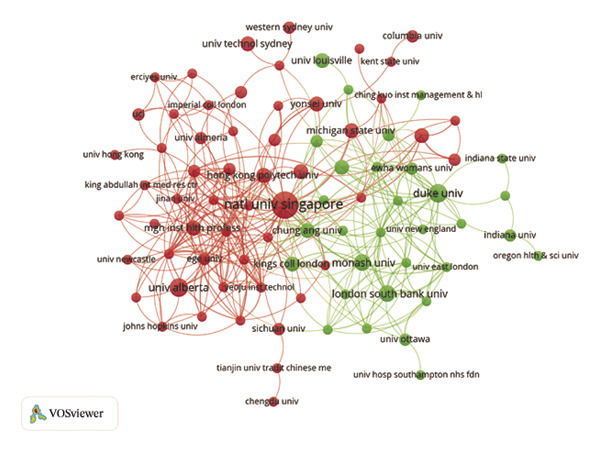
(b)

**Figure:**
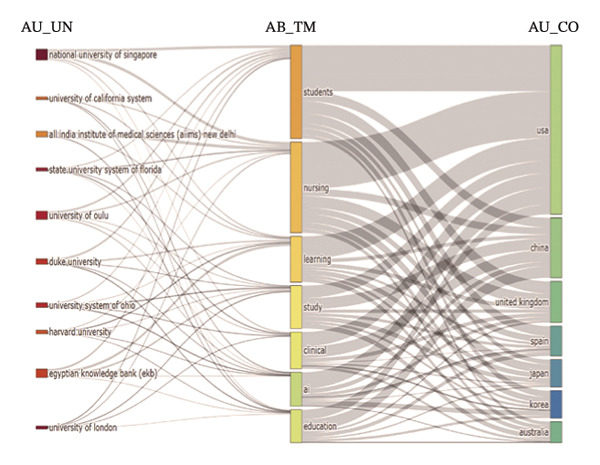
(c)

**Figure:**
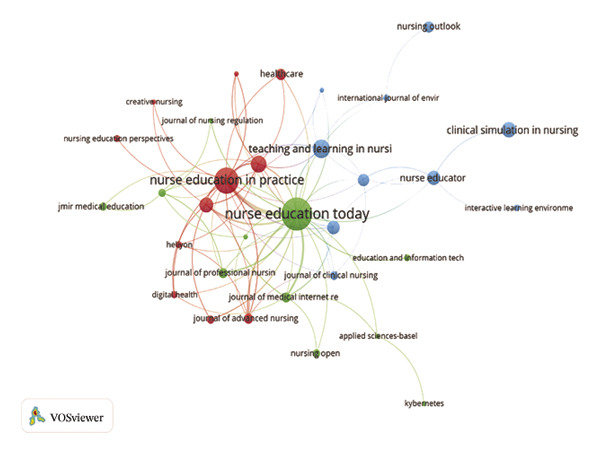
(d)

**Figure:**
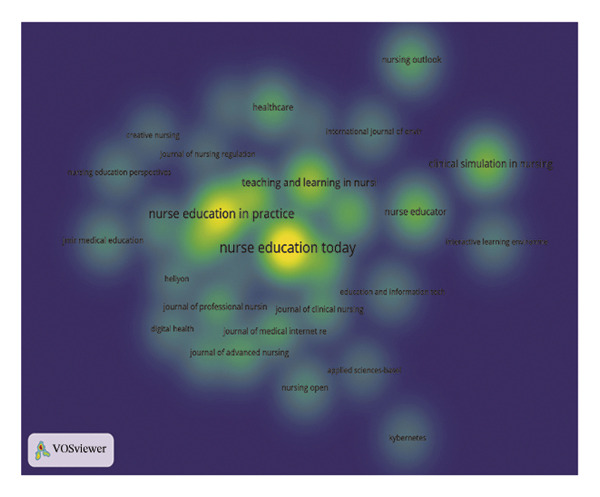
(e)

**Figure:**
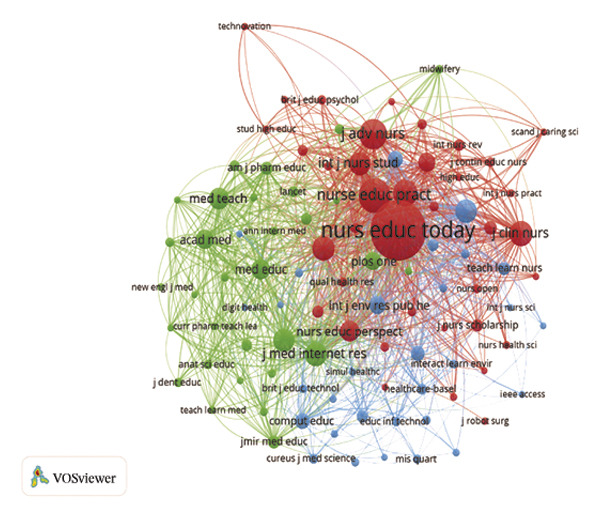
(f)

**Figure:**
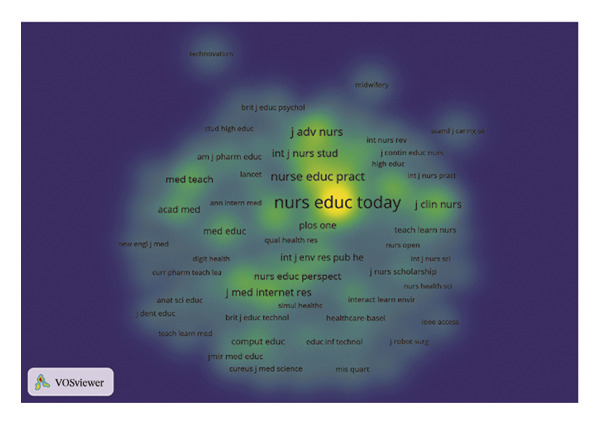
(g)

**Figure:**
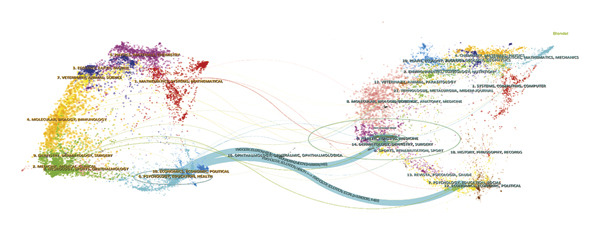
(h)

### 3.3. Analysis of Journals

The journal *Nurse Education Today* was identified as the leading publication in terms of article output, with a total of 47 publications, followed by *Nurse Education in Practice* with 31 publications and *Teaching and Learning in Nursing* contributing 18 publications, as illustrated in Figures 2(d) and 2(e), and Table S4. Furthermore, *Nurse Education Today* also emerged as the most frequently cited journal in the co‐citation analysis, accumulating 794 citations, followed closely by *Nurse Education in Practice* and *J Adv Nurs*, as shown in Figures 2(f) and 2(g) and Table S4. This analysis highlighted the leading role of these journals in the field. Moreover, a dual‐map overlay of the journals further elucidated the citation dynamics between source journals and their co‐cited counterparts, as depicted in Figure 2(h). The two primary citation trajectories indicated that the green curve illustrated a significant flow of citations originating from publications in the psychology/education/health domain and extending to those in the health/nursing/medicine and psychology/education/social domain. This indicated that the research in the field of nursing education was influenced by psychology, pedagogy and health science.

### 3.4. Analysis of Authors

The examination of author collaborations, illustrated in Figure 3(a), highlighted key contributors and their influence within the field of nursing education. Jennie C. De Gagne authored four publications, indicating significant scholarly activity in this area. Furthermore, S. O’Connor held the highest co‐citation count of 50, which indicated his vital role in the field (Figure 3(b) and Table S5).

FIGURE 3(a) An overlay visualization of authors. (b) An overlay visualization of co‐cited authors. (c) Visualization of co‐cited references within the literature. (d) The top 12 references exhibiting significant citation bursts.

**Figure:**
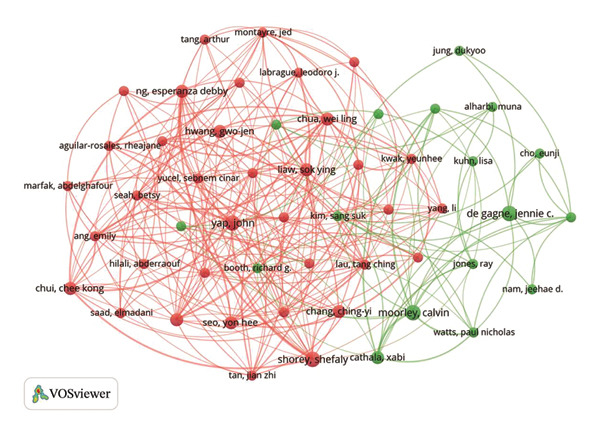
(a)

**Figure:**
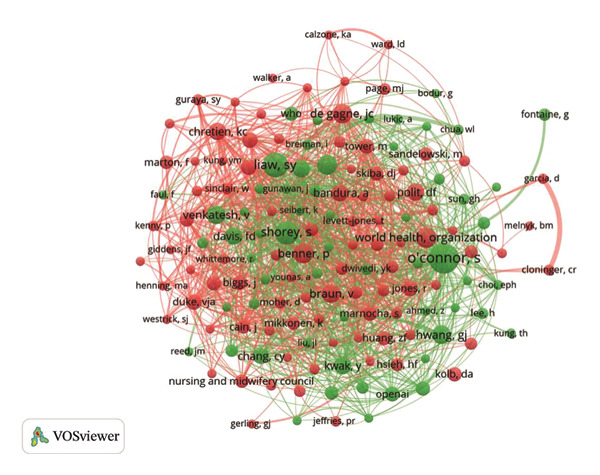
(b)

**Figure:**
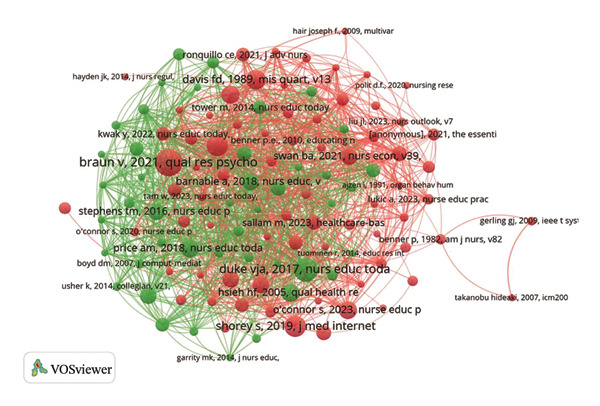
(c)

**Figure:**
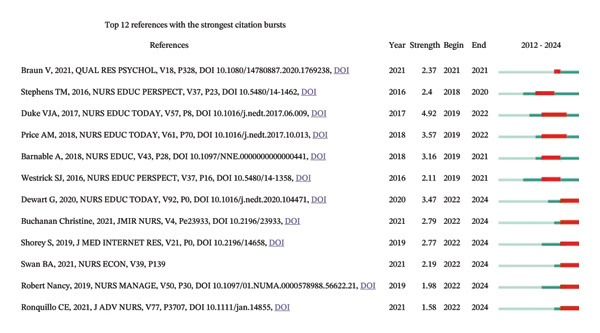
(d)

### 3.5. Analysis of Co‐Cited References

In the evaluation of co‐cited references and those with the most significant citation bursts, the study by Braun V et al. received a total of 18 citations. These data were visually depicted in Figures 3(c) and 3(d), with further details available in Table S6.

### 3.6. Analysis of Keywords

The analysis of keywords revealed emerging trends and key areas within the field, with a significant emphasis on the term “Nursing Education,” which appeared 156 times. The next most frequent keywords were related concepts such as nursing students and AI, as illustrated in Figure 4(a) and detailed in Table S7. The keyword network was categorized into 11 distinct clusters. These clusters highlighted a strong focus on the role of AI in nursing education, which encompassed terms such as AI, nursing students, nursing education, and adapting practice, as depicted in Figure 4(b). Moreover, Figure 4(c) illustrates the evolution of these 11 clusters from 2012 to 2024. The analysis of keywords with citation bursts identified technology, experience, and program development as emerging topics in this area, as shown in Figure 4(d). The thematic map presented in Figure 4(e) highlighted the connections and developmental trajectories among the key topics in this field. Furthermore, the lower right quadrant of this thematic map revealed areas that remain largely unexplored, emphasizing the pressing need for future research to investigate the underlying programs and simulations. Furthermore, the trend theme analysis confirmed “program” and “simulation” as sustained research themes, as demonstrated in Figure 4(f).

FIGURE 4Analysis of keyword visualization. (a) Overlay visualization of keywords. (b) Cluster analysis of keywords illustrated using CiteSpace. (c) The timeline view representation of keyword clusters. (d) The twenty most significant keywords exhibiting the highest citation bursts. (e) Thematic map representing the relationships among keywords. (f) Trend topics in nursing education.

**Figure:**
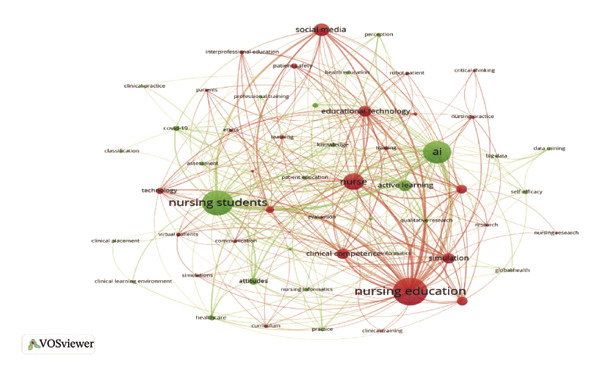
(a)

**Figure:**
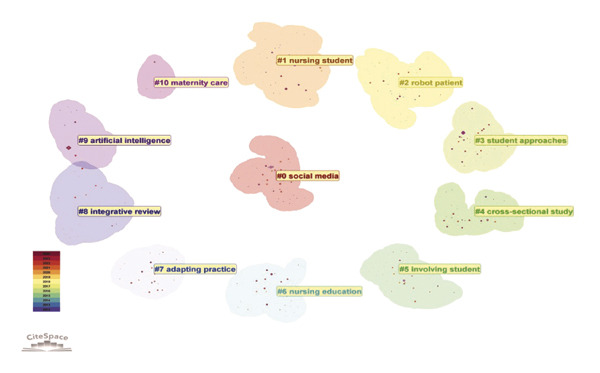
(b)

**Figure:**
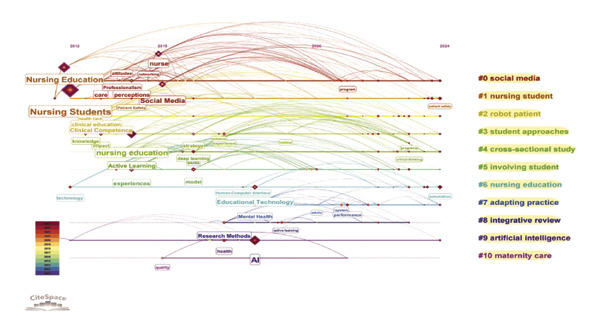
(c)

**Figure:**
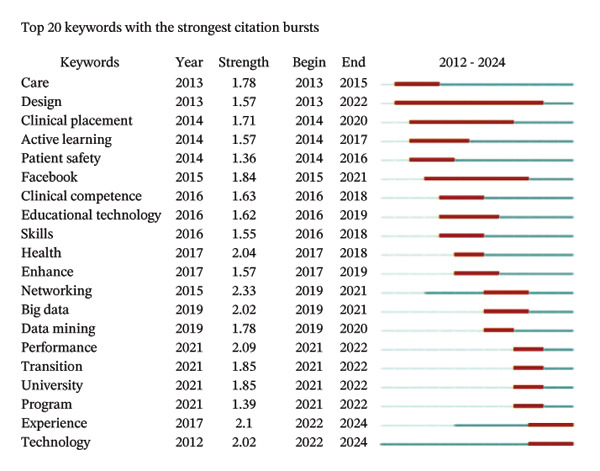
(d)

**Figure:**
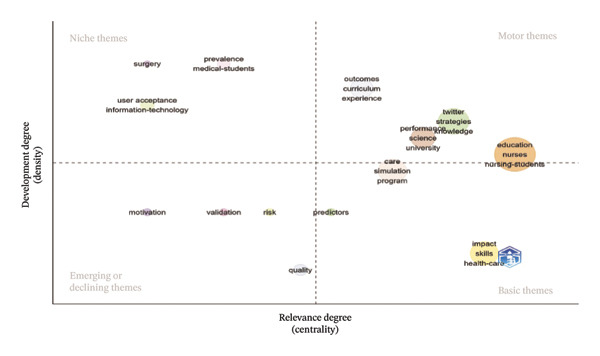
(e)

**Figure:**
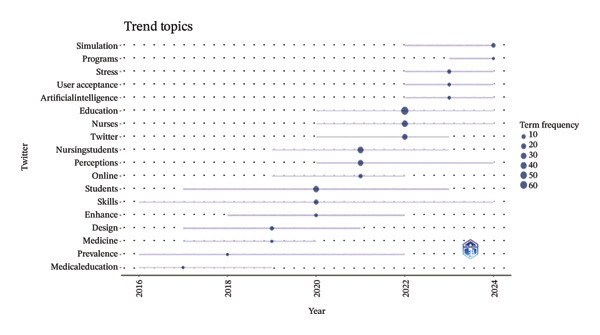
(f)

## 4. Discussion

In this bibliometric analysis, we systematically examined the role of AI in nursing education for students, emphasizing key trends and future directions within the field. Our findings indicated that the United States and China were the leading contributors to this field, highlighting their crucial roles in promoting AI applications in nursing. Moreover, we identified important research hotspots, such as technology, educational programs, and simulation. These hotspots significantly influenced the development of nurse training. This study provides significant perspectives on the current status of AI integration in nursing education and proposes avenues for future exploration and development.

First, the technology has fundamentally transformed nursing education by providing easy access to a wide range of information and resources. With online learning platforms and digital libraries, nursing students can readily obtain evidence‐based practices, clinical guidelines, and the latest research [33, 34]. This level of accessibility not only fosters self‐directed learning but also motivates students to keep up with the latest developments in healthcare. In addition, the incorporation of multimedia resources, including interactive modules, animations, and videos, addresses various learning styles and improves understanding of intricate clinical concepts [35, 36]. By utilizing these technological advancements, nursing educators can create a more inclusive and efficient educational environment that meets the varied requirements of each student.

Building on this technological foundation, technology has significantly improved access to information and has been instrumental in creating innovative educational programs specifically designed for nursing students [37, 38]. For example, numerous nursing schools have embraced blended learning approaches that merge traditional in‐person instruction with online coursework [39]. This hybrid model offers greater flexibility in learning. It also fosters opportunities for students to collaborate on projects and engage in discussions with peers from various backgrounds. Specialized programs focus on essential areas, such as telehealth, informatics, and patient‐centered care, equipping nursing students with the skills to excel in a technologically advanced healthcare environment [11, 40, 41]. By incorporating these programs into their curricula, nursing educators are successfully equipping students to address the complexities of modern healthcare provision and to use technology effectively in their future careers.

Furthermore, the integration of technology extends beyond information access and educational programs. It also plays a vital role in simulation‐based learning, a fundamental aspect of nursing education. Simulation offers students practical experience in a safe and controlled setting [42, 43]. For example, a systematic review by Rabie Adel El Arab reported that the use of generative AI in 25 studies consistently leads to improved critical thinking and clinical satisfaction [44]. Furthermore, another study showed that AI application in nursing research is a dynamic process marked by evolving perceptions and experience [45]. High‐fidelity simulation labs enable nursing students to refine their clinical competencies, make essential decisions, and participate in authentic patient scenarios without the risk of harming real patients. This hands‐on learning method not only improves technical skills but also cultivates vital soft skills such as communication, teamwork, and leadership [46]. Studies indicated that simulation‐based education significantly boosts students’ confidence and competence in clinical environments, which ultimately contributes to improved patient outcomes [47, 48]. Moreover, the integration of augmented reality and virtual reality into simulation training significantly enhances the learning experience by immersing students in realistic scenarios that challenge their problem‐solving abilities and clinical judgment [49].

The integration of technology, innovative educational programs, and simulation in nursing education is crucial for equipping future nurses with the necessary skills to handle the complexities of modern healthcare. These advancements drive improved information access, collaboration, hands‐on experience, and competency‐based learning, directly contributing to a more proficient and assured nursing workforce [50, 51]. It is vital for nursing educators and institutions to continue adopting technological innovations to equip students with the competencies needed to thrive in an increasingly digital healthcare environment [52]. The advancement of nursing education depends on the successful incorporation of these elements, which will lead to enhanced patient care and superior health outcomes for the communities we serve.

The incorporation of AI into nursing education provides substantial advantages while posing considerable obstacles [53]. A primary concern is overreliance on AI that could unintentionally undermine clinical reasoning skills and critical thinking, which are vital in nursing practice [54, 55]. Moreover, ethical issues are also prominent in the role of AI within nursing education [56]. Data privacy and security are critical, especially when handling sensitive patient information. It is crucial that AI systems adhere to ethical standards to safeguard patient confidentiality and teach students how to navigate these complexities. In addition, there is a concern regarding bias in AI algorithms, which could exacerbate existing healthcare disparities if not properly addressed [27, 57]. For instance, the embodiment of algorithmic discrimination has been observed in nursing evaluations. The study by Sharona Hoffman reported how algorithms used in healthcare settings have failed to select racial minorities for beneficial programs and have underestimated health risks for African Americans [58]. In the future, it is imperative to develop clear ethical guidelines and robust training programs that focus on the responsible use of AI in nursing. By addressing these challenges, we can enhance the educational experience for nursing students and ensure they are equipped to deliver equitable and high‐quality care in an increasingly digital healthcare environment.

This bibliometric analysis has a significant limitation due to its exclusive reliance on WOSCC for literature searches. Although WOSCC is well‐regarded for its high‐quality biomedical research and its broad scope of coverage, it may miss relevant studies that are indexed in other databases such as Scopus or PubMed. This oversight could lead to geographical and language biases, as WOSCC has historically provided stronger coverage of English‐language journals from North America and Europe compared to those from other regions or languages. Nevertheless, the rigorous data quality and emphasis on biomedical research within WOSCC enhance the reliability of our results. This provides a strong basis for understanding the significance of AI in nursing education and guiding prospective future research directions.

In summary, our findings reveal a growing interest in the applications of AI, particularly in domains such as enhancing personalized learning, decision‐making, and clinical skills for nursing students. The analysis also highlights the key contributors and institutions at the forefront of this work, indicating a collaborative effort across various disciplines. Moreover, it is crucial that future research investigates both the ethical implications and practical challenges of integrating AI into nursing curricula. The ultimate aim is to advance patient care and enhance educational outcomes across the nursing profession.

## Funding

The authors received no specific funding for this work.

## Conflicts of Interest

The authors declare no conflicts of interest.

## Supporting Information

Supporting Table 1: Retrieval strategy of the role of AI in nursing education.

## Supporting information


**Supporting Information** Additional supporting information can be found online in the Supporting Information section.

## Data Availability

The data that support the findings of this study are available from the corresponding author upon reasonable request.
