# Supplementary material for: Artificial Intelligence in Nursing Education: A Bibliometric Analysis
Source: Nurs Res Pract. 2026 Feb 11;2026:1538554. doi: 10.1155/nrp/1538554 (PMC12892875; doi:10.1155/nrp/1538554)
Supplement: Supplementary file 1 — Supporting Information Additional supporting information can be found online in the Supporting Information section. [file NRP-2026-1538554-s001.docx]

**Supplementary Table 1: Retrieval strategy of the role of AI in nursing education.**

|  | **Search Criteria** | **Records** |
| --- | --- | --- |
| **Records were identified through**  **the WoSCC database** | #1: TS=(“Artificial Intelligence” OR “Computational Intelligence” OR “Machine Intelligence” OR “Computer Reasoning” OR “AI” OR “Computer Vision System” OR “Machine learning” OR robot* OR “robot technology” OR “assistant robot” OR “robot-assisted” OR “computer reasoning” OR “deep learning” OR “sentiment analysis” OR “neural network” OR “expert* system*” OR “natural language processing” OR “decision tree” OR “data mining” OR “Bayesian network” OR “intelligent learning” OR “supervised learning” OR “semantic segmentation” OR “deep network” OR “neural learning” OR “neural nets model” OR “graph mining” OR “ChatGPT” OR “Chatbot” OR “Twitter” OR “TikTok” OR “DeepSeek” OR “Large Language Model” OR “random forest” OR “particle swarm optimization” OR “pattern recognition” OR “genetic algorithm” OR “supervised algorithm” OR “unsupervised algorithm” OR “knowledge-based” OR “casebased reasoning” OR “decision support system” OR “self-organizing map” OR “evolutionary computation” OR “reinforcement learning” OR “clustering” OR “support vector machine”) AND TS=("nursing student*" OR "prelicensure nursing" OR "undergraduate nursing" OR "clinical education" OR "pre-registration nursing" OR "clinical placement" OR "student nurse*" OR "transition to practice" OR “nurs* student” OR “pupil nurs*” OR “nurs* academic” OR “nurs* class*” OR “nurs* course*” OR “nurs* degree”) NOT DT=(Retracted Publication) | 638 |
| **Time span (index date):**  **2012.01.01 to 2024.12.31**  (The search was conducted  on April 18, 2025)  **Languages: English** | #1 | 475 |
| **Excluded Literature** | Meeting Abstract, Letter, Editorial Material, Proceeding Paper, Correction, Data paper,  Retracted Publication | 45 |
| **Remaining Publications** | 392 Articles, 38 Review articles | 430 |

**Supplementary Table 2 Bibliometric analysis overview in VOSviewer**

|  | **Publications** | **Countries** | **Institutions** | **Authors** | **Journals** |
| --- | --- | --- | --- | --- | --- |
| Numbers | 430 | 68 | 775 | 1754 | 194 |

**Supplementary Table 3 Top 10 countries and organization on the field of the role of AI in nursing education.**

| **Rank** | **Country** | **Counts** | **Citations** | **Average Citation per** **Publications** | **Organization** | **Counts** | **Citations** |
| --- | --- | --- | --- | --- | --- | --- | --- |
| 1 | USA | 138 | 1634 | **11.84** | Natl Univ Singapore | 12 | 432 |
| 2 | China | 53 | 478 | **9.02** | Duke Univ | 6 | 75 |
| 3 | England | 34 | 467 | **13.74** | Univ Alberta | 6 | 68 |
| 4 | Australia | 28 | 448 | **16.00** | London South Bank Univ | 5 | 43 |
| 5 | Canada | 23 | 392 | **17.04** | Monash Univ | 5 | 30 |
| 6 | Turkey | 22 | 62 | **2.82** | Chung Ang Univ | 4 | 139 |
| 7 | South Korea | 19 | 449 | **23.63** | Hong Kong Polytech Univ | 4 | 12 |
| 8 | Spain | 17 | 133 | **7.82** | Kings Coll London | 4 | 59 |
| 9 | Japan | 16 | 173 | **10.81** | Mgh Inst Hlth Profess | 4 | 202 |
| 10 | Saudi Arabia | 16 | 90 | **5.63** | Michigan State Univ | 4 | 69 |

**Supplementary Table 4: Top 10 journals and co-cited journals on the field of the role of AI in nursing education.**

| **Rank** | **Journal** | **Counts** | **Citations** | **Co-cited journal** | **Co-citation** |
| --- | --- | --- | --- | --- | --- |
| 1 | Nurse Education Today | 47 | 928 | Nurse Education Today | 794 |
| 2 | Nurse Education In Practice | 31 | 356 | Nurse Education In Practice | 301 |
| 3 | Teaching And Learning In Nursing | 18 | 36 | J Adv Nurs | 240 |
| 4 | Bmc Medical Education | 15 | 246 | J Nurs Educ | 190 |
| 5 | Clinical Simulation In Nursing | 13 | 191 | J Clin Nurs | 170 |
| 6 | Bmc Nursing | 12 | 120 | Nurs Educ | 164 |
| 7 | Nurse Educator | 11 | 102 | Clin Simul Nurs | 161 |
| 8 | Cin-Computers Informatics Nursing | 10 | 87 | J Med Internet Res | 161 |
| 9 | Journal Of Nursing Education | 9 | 81 | Bmc Med Educ | 152 |
| 10 | Healthcare | 7 | 40 | Int J Nurs Stud | 151 |

**Supplementary Table 5: Top 10 authors and co-cited authors on the field of the role of AI in nursing education.**

| **Rank** | **Author** | **Counts** | **Citations** | **Co-cited Authors** | **Citations** |
| --- | --- | --- | --- | --- | --- |
| 1 | De Gagne, Jennie C. | 4 | 58 | O'Connor, S | 50 |
| 2 | Moorley, Calvin | 4 | 35 | Liaw, Sy | 36 |
| 3 | Shorey, Shefaly | 4 | 157 | Shorey, S | 32 |
| 4 | Yap, John | 4 | 206 | World Health, Organization | 26 |
| 5 | Cathala, Xabi | 3 | 26 | Benner, P | 24 |
| 6 | Chang, Ching-Yi | 3 | 143 | Labrague, Lj | 24 |
| 7 | Chua, Wei Ling | 3 | 156 | De Gagne, Jc | 23 |
| 8 | Chui, Chee Kong | 3 | 156 | Braun, V | 22 |
| 9 | Hwang, Gwo-Jen | 3 | 100 | Hwang, Gj | 22 |
| 10 | Liaw, Sok Ying | 3 | 156 | Venkatesh, V | 22 |

**Supplementary Table 6: Top 10 co-cited references on the field of the role of AI in nursing education.**

| Rank | First Author | Year | Journal | DOI | **Citations** |
| --- | --- | --- | --- | --- | --- |
| 1 | Braun V | 2021 | Qual Res Psychol | 10.1080/14780887.2020.1769238 | 18 |
| 2 | Duke Vja | 2017 | Nurs Educ Today | 10.1016/j.nedt.2017.06.009 | 16 |
| 3 | Buchanan Christine | 2021 | Jmir Nurs | 10.2196/23933 | 14 |
| 4 | Shorey S | 2019 | J Med Internet Res | 10.2196/14658 | 14 |
| 5 | Davis Fd | 1989 | Mis Quart | 10.2307/249008 | 13 |
| 6 | De Gagne Jennie C | 2023 | Int J Environ Res Public Health | 10.3390/ijerph20064884 | 12 |
| 7 | Hsieh Hf | 2005 | Qual Health Res | 10.1177/1049732305276687 | 12 |
| 8 | O'Connor S | 2023 | Nurse Educ Pract | 10.1016/j.nepr.2022.103537 | 12 |
| 9 | Price Am | 2018 | Nurs Educ Today | 10.1016/j.nedt.2017.10.013 | 12 |
| 10 | Barnable A | 2018 | Nurs Educ | 10.1097/nne.0000000000000441 | 11 |

**Supplementary Table 7: Top 40 keywords on the field of the role of AI in nursing education.**

| **Rank** | **Keywords** | **Counts** | **Rank** | **Keywords** | **Counts** |
| --- | --- | --- | --- | --- | --- |
| 1 | Nursing Education | 156 | 11 | Research Methods | 22 |
| 2 | Nursing Students | 134 | 12 | Attitudes | 16 |
| 3 | AI | 112 | 13 | Professionalism | 15 |
| 4 | Nurse | 66 | 14 | Technology | 13 |
| 5 | Social Media | 42 | 15 | Covid-19 | 10 |
| 6 | Active Learning | 34 | 16 | Healthcare | 10 |
| 7 | Educational Technology | 32 | 17 | Patient Safety | 10 |
| 8 | Clinical Competence | 29 | 18 | Human-Computer Interface | 8 |
| 9 | Simulation | 29 | 19 | Knowledge | 7 |
| 10 | Mental Health | 22 | 20 | Assessment | 6 |
